# Wearable Devices for Exercise Prescription and Physical Activity Monitoring in Patients with Various Cardiovascular Conditions

**DOI:** 10.1016/j.cjco.2025.02.017

**Published:** 2025-03-03

**Authors:** Tasuku Terada, Matheus Hausen, Kimberley L. Way, Carley D. O’Neill, Isabela Roque Marçal, Paul Dorian, Jennifer L. Reed

**Affiliations:** a School of Life Sciences, University of Nottingham, Nottingham, United Kingdom; b Exercise Physiology and Cardiovascular Health Lab, Division of Cardiac Prevention and Rehabilitation, University of Ottawa Heart Institute, Ottawa, Ontario, Canada; c Institute for Physical Activity and Nutrition, School of Exercise and Nutrition Sciences, Deakin University, Geelong, Victoria, Australia; d School of Kinesiology, Faculty of Professional Studies, Acadia University, Wolfville, Nova Scotia, Canada; e School of Human Kinetics, Faculty of Health Sciences, University of Ottawa, Ottawa, Ontario, Canada; f Department of Medicine, Division of Cardiology, University of Toronto, St Michael’s Hospital, Toronto, Ontario, Canada; g School of Epidemiology and Public Health, Faculty of Medicine, University of Ottawa, Ottawa, Ontario, Canada

## Abstract

As wearable technologies have become increasingly affordable, accessible, and practical, an increasing number of people with cardiovascular disease are beginning to use consumer-grade devices. Common health and wellness metrics reported by wearable devices include heart rate, heart rhythm, and step count, which may afford opportunities to assess cardiovascular conditions, prescribe more personalized exercise for enhanced engagement, and monitor physical activity adherence in patients with cardiovascular disease. This narrative review discusses the application of wearable devices in patients with coronary artery disease, heart failure, atrial fibrillation (AF), cardiac implantable electric devices, and peripheral artery disease in different cardiovascular rehabilitation settings (eg, supervised and home-based). Available literature suggests that, when combined with telemonitoring, wearable devices can increase physical activity participation, thereby improving peak oxygen consumption ($\dot{V}$O_2peak_) and quality of life (QoL) in patients with coronary artery disease, enhancing physical function and QoL in patients with heart failure, and increasing walking capacity and $\dot{V}$O_2peak_ in patients with peripheral artery disease. Wearable devices can also detect AF vs sinus rhythm, guide exercise timing in patients with AF, and monitor safe exercise intensity in patients equipped with cardiac implantable electric devices. Healthcare professionals can promote physical activity by incorporating wearable devices, which can help motivate device users by providing real-time feedback on their behaviours. Commercially available wearable devices have the potential to enhance engagement in physical activity, thereby augmenting the established effects of exercise programs on $\dot{V}$O_2peak_, functional capacity, and QoL in patients with various cardiovascular conditions.

## Résumé

Les technologies portables étant devenues de plus en plus abordables, accessibles et pratiques, un nombre croissant de personnes atteintes de maladies cardiovasculaires (MCV) commencent à utiliser des dispositifs grand public. Les métriques de santé et de bien-être communément rapportées par les dispositifs portatifs comprennent la fréquence cardiaque (FC), le rythme cardiaque et le nombre de pas, ce qui peut permettre d'évaluer les conditions cardiovasculaires, de prescrire des exercices plus personnalisés pour améliorer l'engagement et de surveiller l'adhésion à l'activité physique chez les patients atteints de MCV. Cette revue narrative traite de l'application des dispositifs portables chez les patients atteints de maladie coronarienne (MC), d'insuffisance cardiaque (IC), de fibrillation auriculaire (FA), porteurs de dispositifs électroniques cardiaques implantables (DEI), ou atteints de maladie artérielle périphérique (MAP) dans différents contextes de réadaptation cardiovasculaire (par exemple, supervisée et à domicile). La littérature disponible suggère que, lorsqu'ils sont associés à la télésurveillance, les dispositifs portables peuvent augmenter la participation à l'activité physique, améliorant ainsi la consommation maximale d'oxygène ($\dot{V}$O_2peak_) et la qualité de vie (QdV) chez les patients atteints de MC, améliorant la fonction physique et la QdV chez les patients atteints d'IC, et augmentant la capacité de marche et $\dot{V}$O_2peak_ chez les patients atteints de MAP. Les dispositifs portables peuvent également détecter la FA par rapport au rythme sinusal et guider le choix du moment de l'exercice chez les patients atteints de FA, et surveiller l'intensité de l'exercice en toute sécurité chez les patients équipés d'un DEI. En conclusion, les professionnels de santé peuvent promouvoir l'activité physique en intégrant des dispositifs portables. Ces dispositifs portables peuvent également aider à motiver les utilisateurs en leur fournissant un retour d'information en temps réel sur leurs comportements. Les dispositifs portables disponibles sur le marché ont le potentiel d'améliorer l'engagement dans l'activité physique, augmentant ainsi les effets établis des programmes d'exercice sur le $\dot{V}$O_2peak_, la capacité fonctionnelle et la QdV chez les patients souffrant de diverses affections cardiovasculaires.

Cardiovascular disease (CVD) is the leading cause of death globally, responsible for an estimated 17.9 million deaths each year,^1^ with 30% of ischemic heart disease cases worldwide being linked to physical inactivity.^2^ A supervised cardiovascular rehabilitation (CR) program prevents secondary events and reduces the risk of mortality in patients with CVD.^3^ However, centre-based supervised CR is grossly underutilized, due to several barriers, including lack of physician recommendation, limited accessibility, lack of resources (eg, a lack of caregivers and a long waiting list), and high out-of-pocket expenses.^4^^,^^5^

To overcome barriers to enrollment and participation, CR programs have undergone several changes, including the provision of remote clinical services via real-time, 2-way communication between the patient and the healthcare provider(s) (eg, physiotherapists, kinesiologists, exercise physiologists, nurses, and physicians), using electronic, audio, and visual means (ie, telemedicine).^6^ The shifting of CR programs to remote or hybrid models was accelerated in response to the unprecedented COVID-19 pandemic, which overwhelmed healthcare systems worldwide.^7^ In this era of decentralized CR, healthcare providers are in search of effective and safe ways to implement and monitor physical activity. Patients with CVD also are in need of information about their medical conditions, physical activity levels, and symptoms.^8^

**Figure 1:**
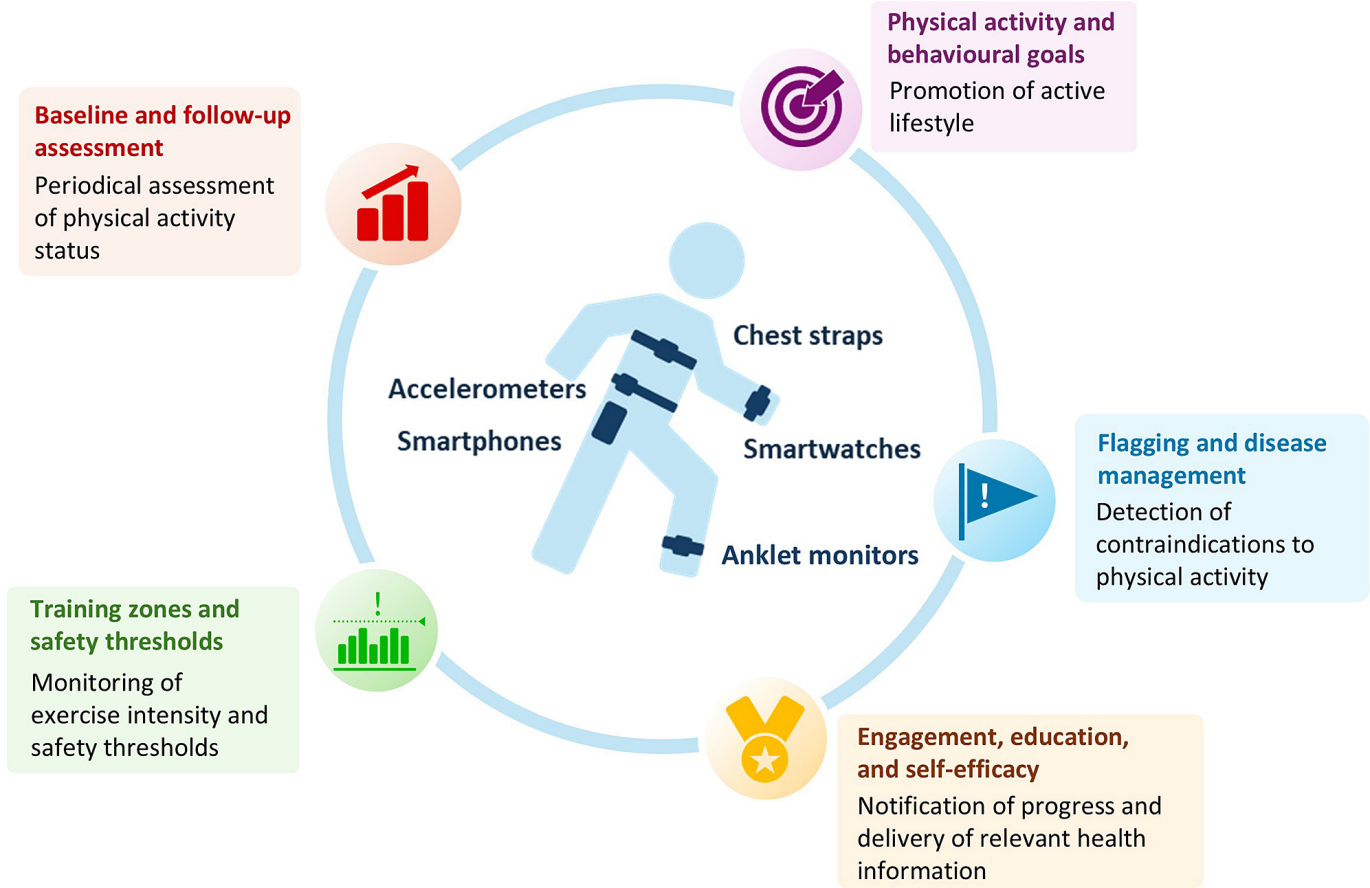
Wearable devices: their measures and applications.Smartphones are not wearable devices, but they are included because they often are used in conjunction with other wearable devices.

Wearable technologies are defined as mobile electronics that can be worn comfortably on the human body^9^ and allow for the continuous monitoring of biometrics in a noninvasive or minimally invasive manner.^10^ Wearable sensors have become increasingly affordable, accessible, and practical,^11^ and an increasing number of people are beginning to use consumer-grade devices to manage their health.^7^ Devices such as fitness trackers, wristbands, patches, smart watches, and biosensors are integrated analytical units equipped with sensitive physical, chemical, and biological sensors capable of noninvasive and continuous monitoring of physiological parameters and physical behaviours.^7^^,^^12^ Common health and wellness metrics measured by wearable devices include heart rate (HR), heart rhythm, respiratory rate, step count, and distance and altitude travelled; in addition, some devices estimate HR variability, blood pressure, oxygen saturation, and peak oxygen consumption ($\dot{V}$O_2peak_).^13^ These measures afford opportunities to screen for cardiovascular conditions, prescribe more-personalized exercise for enhanced engagement and adherence, set attainable physical activity goals (eg, steps and distance), monitor behavioural features (eg, physical activity intensity, duration, and sedentary time),^14^ provide feedback, and flag users regarding irregular physiological responses (Fig. 1).

Systematic reviews have shown that, when used under adequate health professional supervision, wearable devices effectively promote physical activity,^15^^,^^16^ and they contribute to reduction of body mass^17^ in adults with overweight, obesity, and chronic comorbidities. Although such improvements are expected to lower CVD risk,^18^ the effects of wearable devices on CVD risk indicators, including waist circumference, obesity, glycated hemoglobin A1C level, and lipid levels were mixed^16^ or absent^15^ in these reviews. In patients with CVD, systematic reviews and meta-analyses have shown that wearable technology, combined with some form of counselling (eg, tele-coaching, feedback, and personalized goal-setting), is associated with the following changes: increased steps per day and time spent in moderate-to-vigorous intensity physical activity in patients with CVD^19^; increased daily step count and $\dot{V}$O_2peak_, in CR participants vs those who did not receive wearables^20^; and improved cardiorespiratory fitness by nearly 0.5 metabolic equivalents (METs) during the maintenance phase of CR, without increasing the risk for adverse events.^21^

Several studies have discussed the application of wearable technologies to cardiac populations,^21^^,^^22^ including exercise program prescriptions^20^^,^^21^ and behavioural monitoring.^21^ Yet, a knowledge gap in the literature persists regarding the applicability of wearable technologies based on the specific needs of different CVD conditions, such as coronary artery disease (CAD), heart failure (HF), atrial fibrillation (AF), use of cardiac implantable electric devices (CIEDs), and peripheral artery disease (PAD). Incorporating appropriate wearable devices specific to CVDs may enable the following steps: (i) assessment of the severity and prognosis of the conditions; (ii) implementation of more-effective programs; and (iii) more-accurate monitoring of physical activity. The purpose of this narrative review is to summarize the condition-specific applications of available wearable technologies.

## CAD

CAD, which is characterized by atherosclerosis of the coronary arteries, is a leading cause of death globally. CAD manifests in the form of angina, myocardial infarction, exercise intolerance, and rarely, cardiac death.^23^ An increase in physical activity levels and reduction of sedentary behaviour improve prognoses and reduce mortality in patients with CAD, rendering CR a key part of CAD management, along with medical and surgical interventions.^24^ The Canadian Cardiovascular Society (CCS) recommends that patients with asymptomatic CAD (stable ischemic heart disease) accumulate 150 minutes of moderate-to-vigorous intensity physical activity per week.^25^

### Exercise prescription, adherence, and outcomes

In patients with CAD, wearable devices may facilitate and promote physical activity by providing guidance for setting personalized goals, monitoring adherence, and sharing relevant data with healthcare providers to receive feedback.^24^ Further, self-monitoring of HR via commercially available wrist-worn devices (eg, Apple Watch 7 [AW7; Apple, Cupertino, CA] and Galaxy Watch 4 [GW4; Samsung Electronics, Suwon, South Korea]) can provide accurate and real-time feedback of HR during exercise.^26^ Thus, wrist-worn HR monitors can provide guidance regarding a safe exercise intensity for patients with CAD whose ischemic threshold was identified during exercise testing (ie, 10 beats per minute [bpm] below the ischemic threshold^27^). A home-based CR program led by clinical nurse specialists, which integrated pedometers (Yamax Digiwalker NL-2000, Lees Summit, MO) to set graded physical activity tasks and specific goals, and to prompt self-monitoring, improved adherence to physical activity during the first year, compared to the level in the usual-care group.^28^ This improvement was accompanied by greater improvements in quality of life (QoL) measured by the QoL index—cardiac version III. Similarly, a telemonitoring program that set step goals using a triaxial accelerometer (Yorbody accelerometer, Yorbody, Puurs-Sint-Amand, Belgium) added to a standard CR program has been shown to increase $\dot{V}$O_2peak_, which was not observed in the group that received standard CR alone (Table 1).^29^

**Table 1:** Selected randomized controlled trials (RCTs) and systematic reviews (SRs) examining the health benefits of using wearables for different cardiovascular conditions

Study	Device	Design	Intervention	Main findings
**Coronary artery disease**				
Houle et al.^28^ 2012	Pedometer (Yamax Digiwalker NL-200)	RCT	A pedometer-based program with a socio-cognitive intervention to increase patients’ self-efficacy, self-regulation, and self-monitoring; led by a clinical nurse specialist vs usual care (usual advice by the nurse and/or the physician at discharge)	Greater increases in adherence to physical activity (the proportion of participants in the active category at 6, 9, and 12 months in pedometer vs usual-care groups: 75% vs 41%, 68% vs 36%, and 83% vs 55%, respectively) and QoL (24.6 ± 3.9 to 27.7 ± 2.1 points vs 25.7 ± 4.3 to 25.7 ± 4.2 points) in the wearable-device group
Frederix et al.^29^ 2015	Triaxial accelerometer (Yorbody)	RCT	Exercise program in the hospital outpatient rehabilitation centre combined with an exercise training program with telemonitoring support to encourage patients to increase the daily number of steps vs control	A greater increase in $\dot{V}$O_2peak_ in the wearable-device group (24 ± 7 vs 28 ± 6 mL/kg/min vs 22 ± 6 vs 23 ± 6 mL/kg/min)
Hannan et al.^21^ 2019	Pedometers, watches, and smartphones	SR & MA	Wearable devices vs standard care	A greater increase in $\dot{V}$O_2peak_ (MD 1.54, 95% CI: 0.50 to 2.57 mL/kg/min, *P* = 0.004) in the wearable-device group No difference in 6MWT distance
Kaihara et al.^30^ 2022	Accelerometers and pedometers (including software installed on a smartphone)	SR & MA	Wearable devices vs usual care	Greater increases in $\dot{\dot{\dot{V}}}$O_2peak_ (MD 1.65, 95% CI: 0.64 to 2.66 mL/kg/min) and QoL, and a greater reduction in major adverse cardiovascular events (risk ratio 0.51; 95% CI: 0.31 to 0.86) in the wearable-device group
**Heart failure**				
Piotrowicz et al.^34^ 2020	Tele-ECG (also called a telerehabilitation set; Pro Plus Company)	Multicentre RCT	Hybrid comprehensive telerehabitation including the device vs usual care	Greater increases in $\dot{V}$O_2peak_ (0.95, 95% CI: 0.65 to 1.26 mL/kg/min vs 0.00, 95% CI: –0.31 to 0.30 mL/kg/min), 6MWT distance (30.0, 95% CI: 24.7 to 35.3 m vs 20.7, 95% CI: 15.4 to 26.0 m), and QoL (1.58, 95% CI: 0.74 to 2.42 points vs 0.00, 95% CI: –0.84 to 0.84 points) in the hybrid comprehensive telerehabitation intervention
**Peripheral artery disease**				
Normahani et al.^67^ 2018	Wrist-worn activity monitor (Nike Fuelband)	RCT	Feedback-enabled activity monitor in addition to access to supervised exercise program vs supervised exercise program alone	Greater increases in maximum walking distance (82 vs –5 m), claudication distance (63 vs 10 m), and QoL (0.9 vs 0.2 points) in the device group at 6 months and greater increases in maximum walking distance (69 vs 8 m) and claudication distance (38 vs 11 m) at 12 months compared to supervised exercise without devices
Duscha et al.^69^ 2018	Smartwatch (Fitbit Charge)	RCT	Personalized step prescription combined with weekly coaching and text messages vs usual care (no specific lifestyle recommendations but encouraged to follow the guidance provided by physicians).	Greater increases in percent changes in absolute $\dot{V}$O_2peak_ (15.2 ± 4.3 to 18.0 ± 4.8 mL/kg/min vs 14.3 ± 5.4 to 14.5 ± 5.7 mL/kg/min) and claudication onset time (320 ± 226 to 525 ± 252 s vs 252 ± 256 to 231 ± 196 s, in the device group
Thanigaimani et al.^72^ 2022	Wearable activity monitors	SR & network MA	Home exercise programs informed by wearable activity monitors vs home exercise program not informed by wearables vs non-exercise control	Greater increase in walking distance in home-based exercise informed by wearables compared to non-exercise control. No difference between home-based exercise program informed by vs non-informed by wearables.
Gardner et al.^74^ 2014; Gardner et al.^78^ 2011	Anklet step monitor (StepWatch3)	RCT	Home-based exercise session quantified with a step activity monitor combined with feedback vs supervised exercise rehabilitation program with a step activity monitor vs usual care (encouraged to walk more on their own but did not receive specific recommendations about an exercise program)	Home-based program had low attrition and high attendance. Greater improvements in claudication onset time (170 ± 182 vs 104 ± 162 vs 17 ±138 s) and peak walking time (192 ± 190 vs 110 ± 193 vs 22 ± 159 s) in supervised and home-based exercise programs compared to usual care. Between supervised and home-based programs, peak walking time increased more following the supervised program, whereas the 6MWT distance increased more following the home-based program.
McDermott et al.^81^ 2018	Pedometer (Fitbit Zip)	Multicentre RCT	Home-based exercise program consisting of a wearable activity monitor and telephone coaching vs usual care (no study intervention)	Home-based program with activity monitoring did not improve walking performance at 9 months, compared to usual care (MD –8.9 m, 95% CI –26.0 to 8.2).
Waddell et al.^79^ 2024	Smartwatch (Fitbit Charge 4)	RCT	Home-based exercise program consisting of wearable activity monitor-guided walking exercise prescription combined with circuit resistance training vs control (no study intervention)	Home-based program was feasible but did not improve 6MWT distance, maximum walking distance, pain-free walking distance, or QoL at 12 or 24 weeks.

Products are from the following companies: Charge, Charge 4, and Zip (all from Fitbit, San Francisco, CA); Nike Fuelband (Nike, Beaverton, OR) StepWatch3 (Cyma, Mountlake Terrace, WA); telerehabilitation set (Pro Plus, Warsaw, Poland); triaxial accelerometer (Yorbody, Puurs-Sint-Amand, Belgium); Yamax Digiwalker NL-200 (Yamasa Tokei Keiki Co, Ltd, Tokyo, Japan).

CI, confidence interval; ECG, electrocardiogram; MA, meta-analysis; MD, mean difference; QoL, quality of life; $\dot{V}$O_2peak_, peak oxygen consumption; 6MWT, 6-minute walking test.

Systematic reviews and meta-analyses (n = 11 randomized controlled trials, with a total of 1356 participants^30^; and n = 9 studies, with a total of 1352 participants^21^) show that, during and following CR, wearable devices, combined with healthcare expert guidance and feedback, increase $\dot{V}$O_2peak_ and QoL, and they reduce the risk of major adverse cardiovascular events (Table 1).^21^^,^^30^ Digital health interventions integrating a smartphone application, smartwatch, and blood pressure monitoring also resulted in a 52% lower risk of all-cause 30-day readmissions, compared to standard-of-care in patients with acute myocardial infarction.^31^ These positive outcomes may be mediated by improved patient engagement in self-management and health monitoring, facilitated by wearable devices. Although the cost-effectiveness of use of wearable devices in patients with CAD remains unclear, a large-scale trial—ITELEWEAR-CR—is currently under way to assess the efficacy, efficiency, safety, and cost-effectiveness of a telerehabilitation program using wearable sensors in patients with CAD.^14^

### Summary

The existing literature supports the incorporation of commercially available wrist-worn devices and pedometers to guide and monitor exercise programs in patients with CAD. When combined with established exercise programs or CR, commercially available wearable devices (eg, Apple, Galaxy, and Garmin [Kansas City, MO] smartwatches) promote physical activity engagement, which in turn increases $\dot{V}$O_2peak_ and QoL and reduces the risk of major adverse cardiovascular events in patients with CAD.

## HF

HF is a complex syndrome resulting from structural or functional impairment of ventricular filling or ejection of blood, which leads to marked changes in functional status and QoL. The 2 main classifications of HF based on left ventricular ejection fraction are HF with reduced ejection fraction (left ventricular ejection fraction ≤ 40%) and HF with preserved ejection fraction (left ventricular ejection fraction ≥ 50%). Exercise training and CR are strongly recommended by the CCS for the management of HF, specifically to improve exercise and functional capacity and QoL.^32^

### Detection and assessment for exercise prescription

The advancement that has been made in wearable technology offers an opportunity to assess the severity and prognosis of HF and provides objective measures that can be used to monitor progress or regression in HF status. Monitoring peripheral and pulmonary edema in HF is important for identifying early signs of HF decompensation, a contraindication for engaging in physical activity. The BodiGuide anklet (BodiGuide, Bellevue, WA) has been shown to be a feasible method to use to recognize clinically relevant changes in peripheral edema and decompensation in HF.^33^

### Exercise adherence and outcomes

Few trials have integrated wearables as part of telerehabilitation in HF. For example, the Telerehabilitation in Heart Failure Patients (TELEREH-HF) trial incorporated a tele-electrocardiogram (ECG; also called a telerehabilitation set, Pro Plus, Warsaw, Poland) and showed greater increases in 6-minute walking test (6MWT) distance, $\dot{V}$O_2peak_, and QoL in the telerehabilitation group vs the usual-care group (Table 1).^34^ However, no differences were observed in mortality and hospitalization over a follow-up period of 14-26 months. Additionally, the Canagliflozin: Impact on Health Status, Quality of Life and Functional Status in Heart Failure **(**CHIEF-HF) study used the Fitbit Versa 2 (Fitbit, San Francisco, CA) to collect daily step counts over a period of 12 weeks and showed that increased mean daily step count was associated with improved total symptom and physical limitation scores, and QoL as measured by the Kansas City Cardiomyopathy Questionnaire.^35^ Of the 448 participants included in the analysis, 426 (95%) had sufficient data (> 7 days of wear data over a 2-week period), indicating high adherence to use of the wrist-worn device.^35^ These results highlight that exercise programs incorporating wrist-worn wearables may lead to increased step counts and improvement in symptoms, physical limitations, and QoL in patients with HF.

Several commercially available wrist-worn devices (i.e., Withings [Issy-les-Moulineaux, France], Fitbit Charge, Garmin vívofit, and Garmin vívofit 3) demonstrated no systematic differences in step counts, compared to counts taken using the ActiGraph wGT3X-BT (ActiGraph, Pensacola, FL) in HF patients.^36^ In addition to use of these wrist-worn devices, the integration of automated text messaging, online or application group chats, or video calls can provide additional support and reminders to help patients adhere and comply to an exercise prescription, increase their step counts, and improve their health outcomes. Peng and colleagues used an instant messaging platform to communicate via text-based, audio, or video calls to support patients with HF who were undergoing exercise telerehabilitation.^37^ Compared to the control group, those who received telerehabilitation improved their 6MWT distance (from 407 ± 12 to 420 ± 10 minutes vs from 406 ± 12 to 407 ± 13 minutes, *P* < 0.01) and QoL (from 48.8 ± 12.2 to 49.2 ± 12.4 points vs from 49.4 ± 12.3 to 43.1 ± 8.8 points, *P* < 0.05).^37^

### Summary

HF symptom monitoring using wearable devices may allow for safer prescription of exercise, by identifying those who have contraindications to engaging in physical activity. Although only limited options are currently available to provide a robust exercise prescription using wearables for adults living with HF, the use of wrist-worn devices, combined with smartphone messaging, video call applications, and e-mails, to provide support and feedback on exercise prescription, can have a positive effect on functional capacity and QoL in patients with HF.

## AF

AF, which constitutes both the most common sustained cardiac arrhythmia and a global epidemic, afflicts more than 37 million people worldwide.^38^ This burdensome medical condition is associated with variable and occasionally disabling symptoms, such as palpitations, shortness of breath, light-headedness, fatigue, and reduced exercise tolerance.^39^ The CCS suggests regular moderate-intensity aerobic (≥ 200 minutes per week), resistance (2-3 days per week), and flexibility (≥ 10 minutes per day at least 2 days per week in those aged ≥ 65 years) training.^39^ An understanding of an individual’s unique HR control issues may lead to more-effective exercise prescriptions.

### Detection and assessment for exercise implementation

For patients with paroxysmal AF, exercise may be performed while they are in sinus rhythm, to avoid breathlessness and discomfort. For patients with persistent or permanent AF, exaggerated HR response may be monitored, to reduce the risk of shortness of breath or dizziness. Several wearable devices have regulatory approval to detect AF (and other arrhythmias). The most widely used AF-detection algorithms (eg, those of the Apple Watch [Apple], Fitbit [Fitbit], and Gear Fit2 [Samsung] smartwatches) use optical detection of photoplethysmographic (PPG) signals to detect an irregular pulse; the specificity is increased by requiring several periods of irregular pulse to occur before the user is notified of a possible arrhythmia.^13^ These alerts do not diagnose AF, and screening of AF through PPG has been shown to have variable accuracy, depending on the algorithms and wearables used.^7^ However, in the large-population Fitbit Heart Study (n = 455,699), a novel PPG software algorithm for wearable Fitbit devices had a high positive predictive value for concurrent AF and identified participants likely to have AF on subsequent ECG patch monitoring.^40^

Some wearable devices have regulatory approval for ECG single- and multi-lead acquisition and accompanying automated algorithms for rhythm classification (eg, KardiaMobile [AliveCor, Mountain View, CA], Zephyr BioHarness 3.0 [Medtronic, Dublin, Ireland], and Vivalink [Vivalink, Campbell, CA]).^41^ These AF classification algorithms are proprietary to each manufacturer and vary in performance, depending on the population using the wearable device.^13^ Other technologies for AF detection include commercially available smartwatches with PPG and ECG capabilities (eg, Apple, Samsung, and Withings smartwatches).^41^ Emerging research has shown equivalent diagnostic performance of PPG and single-lead ECG proprietary AF detection algorithms in smartphone apps vs the gold-standard 12-lead ECG.^42^

### Exercise prescription

Wearables offer several features (eg, measurement of HR and $\dot{V}$O_2peak_, activity tracking) that can be used to assess the frequency, intensity, time, and type principles of exercise prescription. HR is perhaps the most inaccurate or unreliable measurement to use to prescribe and monitor exercise intensity in those living with AF, given their unique HR control issues. A study testing 6 commercially available wearables (Apple Watch Series 3 [Apple], Charge HR [Fitbit], Blaze Smart Fitness Watch [Fitbit], Polar A360 [Polar, Kempele, Finland], vivosmart [Garmin], and Jabra Sport pulse headphones [Jabra, Copenhagen, Denmark]) found a mean absolute difference between ECG-measured HR vs HR as measured by the wearables of 7 ± 12 bpm at rest and 28 ± 23 bpm at peak exercise in patients with persistent AF.^43^ Al-Kaisey et al. reported that smartwatches underestimate HR in those with persistent AF, particularly at HR ranges that are > 100 bpm^44^; peak HR measured during a treadmill stress test showed greater absolute mean differences between wearables and ECG recordings (Apple Watch Series 3, 21 bpm; Fitbit Charge, 30 bpm; Fitbit Blaze, 30 bpm; Polar A360, 29 bpm; and Garmin vívosmart, 36 bpm).^43^ Similarly, in patients with permanent AF, HR does not seem to provide an accurate measure of exercise intensity, given their weak association^45^; this is concerning for those with AF who may use these inaccurate HRs to guide their exercise intensity. Nonetheless, HR monitors may still play an important role in guiding patients on when to exercise and when to slow down.

### Summary

Wearable devices offer an opportunity for patients to detect when they are in AF vs sinus rhythm in their own free-living environment. Yet, users should be aware of the limitations regarding the accuracy and reliability of their HR and heart rhythm assessments. To monitor physical activity levels of patients with AF, other forms of wearable devices (eg, pedometers and accelerometers) may provide more-accurate assessments.^45^ Healthcare professionals and users also should be aware that patients with AF who use wearable devices may demonstrate preoccupation with their cardiovascular symptoms, engage in excessive symptom monitoring, and report more concerns.^46^

## CIEDs

CIEDs—permanent pacemakers, implantable cardioverter defibrillators (ICDs), and cardiac resynchronization therapies (CRTs)—are essential to manage and treat heart rhythm disorders.^47^ Remote monitoring of CIEDs is a class 1A recommendation to monitor patients’ cardiac rhythm.^48^ Some modern CIEDs are also equipped with built-in accelerometers, which enable rate-responsive pacing (eg, adjusting HR according to oxygen demands) and automatic collection and storage of the device-measured physical activity when a rate sensor is activated.^49^ In patients with CIEDs, exercise and physical activity have been shown to be safe and effective in increasing cardiorespiratory fitness and to reduce all-cause mortality.^50^

### Exercise monitoring and outcomes

Wearable devices recently have been recommended to patients equipped with CIEDs, to continuously monitor their physical activity at home and during their daily life.^51^ These include smartwatches and mobile phones, which are capable of ECG recording, HR monitoring, activity and sleep tracking, fall detection, and medication reminders.^52^ Smartphones can detect exercise-induced arrhythmias, as described above (eg, via ECG), and chronotropic incompetence (ie, inability to increase HR during exercise), and they can provide continuous visual presentation of HR to help patients avoid having it reach the thresholds for anti-tachycardia pacing and shocks, in those with ICDs or CRT with defibrillators.^53^ These functions may support safe engagement in exercise. Two trials assessing the safety and efficacy of an 8-week (5 days/week) home-based exercise program in patients with CIEDs demonstrated that the integration of mobile phones into home-based exercise is safe (eg, no adverse events occurred) and feasible (> 90% adherence), and is associated with improvement in $\dot{V}$O_2peak_ (16.1 ± 4.0 vs 18.4 ± 4.1 mL/kg per minute, *P* < 0.001), 6MWT distance (428 ± 93 vs 480 ± 87 meters, *P* < 0.001), and QoL (79.0 ± 31.3 vs 70.8 ± 30.3 points, *P* < 0.001—lower scores indicate better QoL).^54^^,^^55^ An important finding is that 93% of patients reported that the telerehabilitation program stimulated their exercise, and 87% increased their everyday physical activities.^55^

Traditional external accelerometers have been shown to be an accurate tool to access and improve physical activity levels in patients with CIEDs.^56^ Further, growing research has explored the usefulness of modern CIEDs with built-in accelerometers for tracking physical activity levels.^57^ Physical activity measured by CIED has been shown to correlate strongly with validated, externally worn accelerometers (intra-individual correlations, *r* > 0.7).^58^ These studies highlight the potential use of CIED-embedded activity monitors to capture physical activity levels. Nonetheless, further research is needed to test the accuracy and generalizability of these physical activity measures, as differences in signal processing across manufacturers may influence the physical activity levels recorded.^57^Although patients equipped with CIEDs are interested in accessing their physical activity data and device-related health information,^59^ the technical language of current CIED-generated reports (eg, interrogation reports) limits their accessibility. More accessible and personalized reports in plain language will provide an enhanced opportunity to integrate technology to educate patients on healthy behaviours and better self-management.

Healthcare professionals and patients equipped with CIEDs may take advantage of wearable devices to monitor HR and heart rhythm for safety during exercise. Previous studies have shown that wearable devices (often wrist watches) are practical resources to monitor exercise intensity (eg, moderate-to-vigorous intensity exercise) in patients equipped with ICDs and CRTs.^51^ With an advancement in the technology of CIEDs, they may be used as an adjunct therapy to provide more accessible and valuable information to patients and healthcare providers, especially when their use is combined with additional information from externally worn devices.

### Summary

CIEDs combined with wearable devices may afford safe and effective exercise prescriptions for patients with CIEDs. Yet, further research is warranted, as the literature examining exercise and wearable devices in patients with CIEDs is limited, and existing trials carry the limitations of small sample sizes or retrospective designs. The reliability and validity of wearable devices in patients with CIEDs also need to be established.

## PAD

PAD is caused by atherosclerotic occlusion of the arteries to the lower extremities. Patients with PAD are often burdened with poor QoL,^60^ intermittent claudication (ie, muscle pain in the legs while walking that resolves when walking is discontinued),^61^ and high risk of amputations^61^and mortality.^62^ Despite these conditions, patients with PAD are undertreated compared to those with CAD.^60^^,^^62^ The recommended first-line treatments for PAD include cardiovascular risk reduction through exercise,^63^ preferably in the form of walking to increase walking capacity.^64^ Because patients with intermittent claudication report difficulty conducting prolonged continuous exercise due to muscular pain, they are advised to perform intermittent bouts of walking to induce moderate-to-high claudication pain alternating with periods of rest for at least 30 minutes, 3 times per week.^61^^,^^64^

### Exercise prescription, adherence, and outcomes

Supervised exercise programs have been shown to improve pain-free walking time and distance, and QoL in patients with PAD^65^^,^^66^; these effects may be enhanced by incorporating wearable devices. For example, when added to a 12-week supervised exercise program, commercially available wrist-worn activity monitors (Nike+ FuelBand, Nike, Beaverton, OR) that allow for motion quantification, personalized goal-setting, real-time feedback on progression, and comparison of activity with peers or a broader community of users have been shown to increase maximum walking distance and claudication distance at 6 and 12 months, and QoL at 6 months, more than supervised exercise without devices (Table 1).^67^ In a veteran population living with claudication, the addition of a step-tracking device (Fitbit [Fitbit]) to a supervised exercise program increased walking distance (mean overall changes in steps, 3492, 95% CI 2661-4322 to 4502, 95% CI 3636-5367 at 5 months), especially among those who had less-severe claudication, although a decline in use of activity trackers over time was observed.^68^ Following completion of CR, setting progressive step-count goals, combined with weekly tips to understand PAD and self-care using a Fitbit Charge (Fitbit) device has been shown to increase $\dot{V}$O_2peak_ and claudication onset time, as compared to usual care over 12 weeks (Table 1).^69^

Despite their well-established effectiveness, supervised exercise programs are heavily underutilized in PAD,^70^ often due to limited accessibility.^71^ When supervised exercise programs are not available or feasible, a structured home-based exercise program including behaviour-change techniques is recommended.^64^ Monitored home-based exercise programs have been shown to be effective in improving walking capacity in patients with PAD^72^, ^73^, ^74^; the improvements in maximum and pain-free walking distance are similar to those observed following supervised exercise programs.^75^ The available evidence suggests that commercially available wearable devices may play a crucial role in the successful implementation of home-based exercise programs in patients with PAD, by supporting patient monitoring, education, self-efficacy, goal-setting, and training plans, to increase adherence.^75^^,^^76^ For example, a smartphone application synchronized with a wearable activity tracker to provide motivation and information to improve behaviour skills can promote physical activity in patients with PAD.^77^ When step-count monitor (StepWatch3, Cyma, Mountlake Terrace, WA) information (eg, achievement and progress) is discussed and feedback provided, large-scale RCTs (N = 100+) have shown that a 12-week home-based exercise program induced similar improvements to those produced by a supervised exercise program in claudication onset time, peak walking time, vascular function, inflammation, daily average walking cadence, and 6MWT distance.^74^^,^^78^ Systematic reviews also support the enhanced benefits of home-based interventions that include wearable devices—in maximum walking distance, claudication distance, 6MWT distance,^79^ steps per day, $\dot{V}$O_2peak_, and QoL—compared to those with standard care and/or supervised exercise (Table 1).^72^^,^^80^ An important point to note is that the effectiveness of incorporating wearable devices may be limited to studies that have a short duration. as a multicentre randomized trial showed that a home-based exercise intervention incorporating wearable technology (Fitbit Zip [Fitbit]) and telephone coaching did not improve 6MWT distance at 9 months, compared to usual care.^81^ This lack of improvement may also be attributed to low adherence to use of the device, as 10% of 99 participants had no Fitbit data, and 56.5% of Fitbit data was missing from the remaining participants.^82^ In addition, home-based CR combined with wearables may only be effective for walking interventions. A study led by Waddell et al. showed that home-based CR incorporating the Fitbit Charge 4 (Fitbit) and circuit resistance exercise did not improve walking capacity or QoL.^79^

### Summary

The available evidence suggests that commercially available wearables can be used to augment the effects of supervised exercise programs on walking capacity and QoL in patients with PAD. After CR graduation, the provision of progressive step-count goals is associated with further increases in walking capacity and $\dot{V}$O_2peak_. A program that combines wearable activity monitors with behavioural change constructs (eg, feedback, goal-setting, incentives, and support) to guide and educate patients may be a practical and effective approach to improving walking distance and QoL in patients with PAD. However, the long-term effects of adding wearable devices to home-based programs and their benefits in those with more advanced claudication require further investigation. In addition, although a daily step count is the most clinically relevant and appropriate metric to evaluate daily activity levels in patients with PAD,^83^ pedometer activity measures remain to be validated in patients with PAD.^84^ Given the positive association between exercise intensity and walking distance and $\dot{V}$O_2peak_ in PAD,^85^ other wearable devices, such as HR monitors, also may be incorporated to guide exercise intensity to produce greater benefits.

## Discussion

Commercially available wearable technologies may offer more individualized exercise prescriptions and encourage safe engagement in physical activity in patients with CVDs. Additionally, the fact that many wearable devices can report data remotely^68^ may provide healthcare practitioners with enhanced opportunities to intervene, thereby increasing patients’ program adherence and compliance. When combined with motivational feedback from healthcare practitioners, wearable devices are expected to improve a wide range of health measures in patients with CVDs, including daily physical activity level, cardiorespiratory fitness, functional capacity, and QoL. Figure 1 summarizes the wearable devices evaluated in this review and their potential applications.

**Figure 2:**
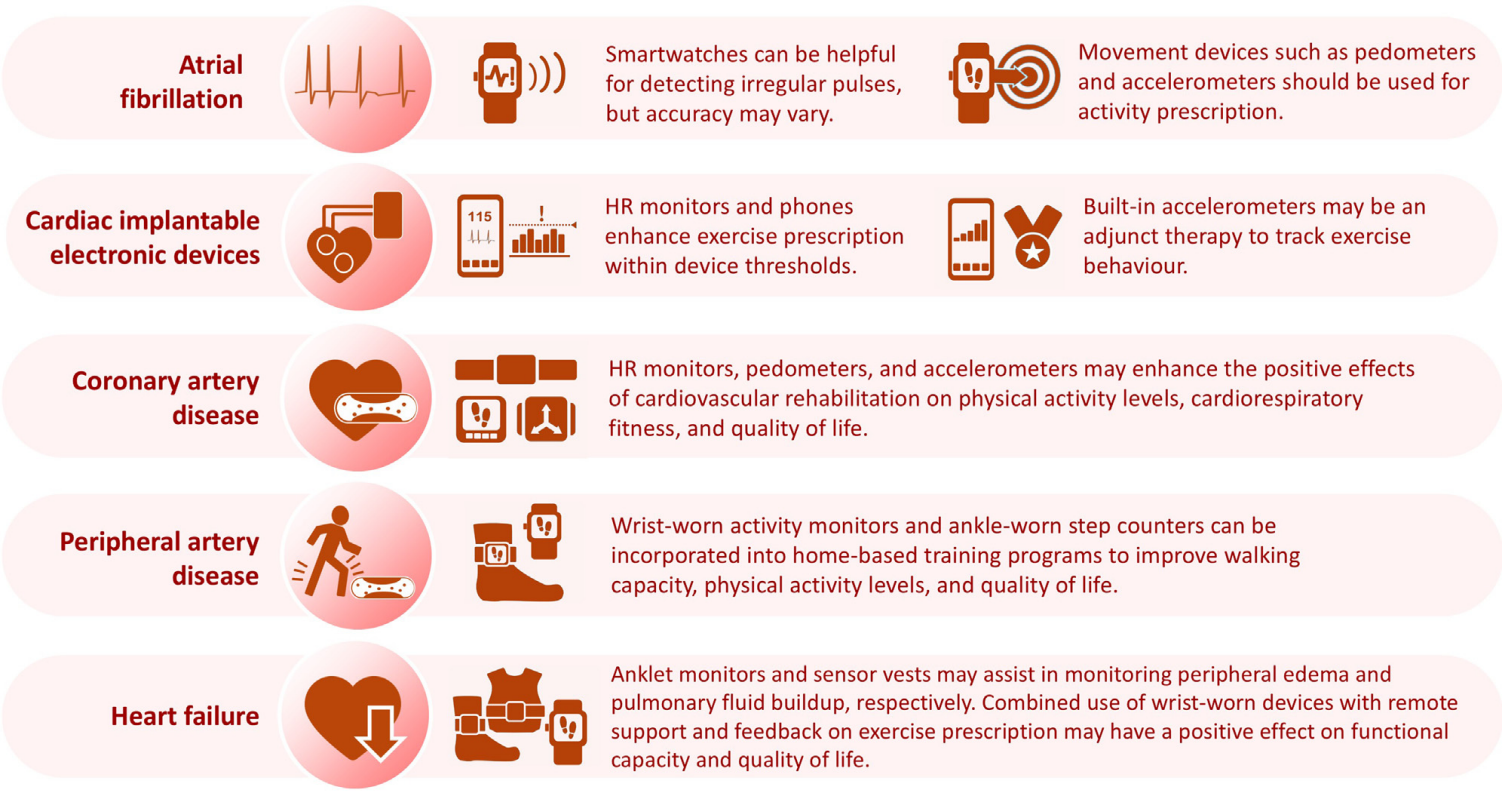
Cardiovascular condition-specific applications of wearable devices. AF, atrial fibrillation; ECG, electrocardiogram; HR, heart rate.

An important point to note is that the usefulness of device-guided exercise prescription and subsequent device-monitored physical activity is dependent on adherence to device use. In a systematic review examining adherence to an activity-monitoring device among adults with any cardiovascular conditions (n = 10 studies), the mean adherence level was 59.1%, with a wide range of variation—39.6%-85.7%.^86^ Selecting wearable devices that best match the unique need specific to a patient’s cardiovascular condition may increase adherence. Our review showed that wrist-worn wearable devices are the most widely studied and adopted devices among patients with various cardiovascular conditions. When combined with telemonitoring, use of these devices can increase physical activity participation, thereby improving $\dot{V}$O_2peak_ and QoL in patients with CAD, enhancing physical function and QoL in those with HF, and increasing walking capacity and $\dot{V}$O_2peak_ in patients with PAD. Wearable devices also may detect AF vs sinus rhythm and guide exercise timing in patients with AF, and monitor safe exercise intensity in patients equipped with ICDs and CRTs (Fig. 2).

Digital healthcare, including use of wearable devices, can make healthcare systems more efficient and sustainable, facilitating delivery of good quality, affordable, and equitable care.^87^ Although the use of technology by cardiac patients is increasing rapidly, with more than 80% of patients holding positive perspectives toward technology and its applications,^88^ the level of use of wearable devices is significantly lower among those with CVD (18%), compared to the level among adults without CVD (29%).^89^ Considering that a lack of digital literacy may impede the equitable implementation of digital technologies,^90^ adequate education of practitioners and patients may facilitate the effective and longstanding use of wearable technology in cardiovascular prevention and rehabilitation. Further, because wearable-based diagnosis algorithms can be susceptible to false-positive signals, leading to enhanced anxiety and increased use of healthcare resources, such as other analysis-based diagnostics, algorithms should meet quality-control standards.^12^ The clinical assessment of all wearable device data should begin with careful consideration of potential device–patient interface issues, motion artifacts, symptom presence/absence, and the frequency of repeated occurrences.^13^ Improving the digital literacy of patients and care providers to better their understanding of the limitations of wearables and how to differentiate false-positive signals from actual contraindications to exercise would facilitate the safe implementation of exercise and promote more-active lifestyles.

### Conclusions

Wearable devices are valuable for prescribing and monitoring physical activity in patients with various CVDs across different CR settings (eg, supervised and home-based). By incorporating wearable devices that align with the needs of specific cardiovascular conditions, the established benefits of exercise programs on cardiorespiratory fitness, walking capacity, and QoL may be enhanced in patients living with various cardiovascular conditions.
